# Research on Low-Cost High-Viscosity Asphalt and Its Performance for Porous Asphalt Pavement

**DOI:** 10.3390/polym16111489

**Published:** 2024-05-24

**Authors:** Lei Xia, Qidong Su, Lingyan Shan, Fulong Li, Dongwei Cao, Jie Lu

**Affiliations:** 1 School of Materials Science and Engineering, Chang’an University, Xi’an 710061, China; dwcphd@163.com; 2 Research Institute of Highway Ministry of Transport, Beijing 100088, China; 17556872697@163.com (Q.S.); lujie1994rioh@163.com (J.L.); 3 Beijing Evolyzer Co., Ltd., Beijing 102600, China; fulongli6666@126.com

**Keywords:** porous asphalt pavement, high-viscosity asphalt, high performance, modification mechanism, cost-effective

## Abstract

To develop a cost-effective, high-viscosity asphalt for porous asphalt pavement, we utilized SBS, tackifier, and solubilizer as the main raw materials, identified the optimal composition through an orthogonal experiment of three factors and three levels, and prepared a low-cost high-viscosity asphalt. We compared its conventional and rheological properties against those of rubber asphalt, SBS modified asphalt, and matrix asphalt, employing fluorescence microscopy and Fourier transform infrared spectroscopy for microstructural analysis. The results indicate that the optimal formula composition for high-viscosity asphalt was 4–5% styrene-butadiene-styrene (SBS) + 1–2% tackifier +0–3% solubilizer +0.15% stabilizer. The components evenly dispersed and the performances were enhanced with chemical and physical modification. Compared with SBS modified asphalt, rubber asphalt, and matrix asphalt, the softening point, 5 °C ductility, and 60 °C dynamic viscosity of high-viscosity asphalt were significantly improved, while the 175 °C Brookfield viscosity was equivalent to SBS modified asphalt. In particular, the 60 °C dynamic viscosity reaches 383,180 Pa·s. Rheological tests indicate that the high- and low-temperature grade of high-viscosity asphalt reaches 88–18 °C, and that high-viscosity asphalt has the best high-temperature resistance to permanent deformation and low-temperature resistance to cracking. It can save about 30% cost compared to commercially available high-viscosity asphalt, which is conducive to the promotion and application of porous asphalt pavement.

## 1. Introduction

With the advent of the automobile era, the challenges to road traffic safety have intensified. According to investigation results concerning highway accidents during rain, the accident rate on rainy days is approximately eight times higher than on sunny days, featuring a high incidence of severe accidents, such as consecutive rear-end collisions and hydroplaning. Furthermore, the noise generated by the interaction between the road surface and tires during high-speed travel significantly impacts the living environment of residents along these routes [1]. In this context, porous asphalt pavement has emerged as the optimal solution for mitigating frequent rain-related accidents, owing to its superior noise reduction, skid resistance, water mist suppression, enhancement of driving safety in wet conditions, and reduction in surface runoff and water pollution [2,3,4,5]. Porous or permeable asphalt pavement is characterized by asphalt mixtures with large pores, allowing surface rainwater to penetrate the structural layer and infiltrate into the base layer [6,7,8]. Compared to conventional pavement, the cushion layer of porous asphalt pavement requires high permeability, consisting primarily of graded crushed stone, gravel, or a mixture thereof, forming various granular cushion layers. The soil foundation beneath the cushion layer typically comprises sandy soil with excellent permeability, meeting the structural requirements for pavement [9,10,11]. Water damage is widely recognized as the primary cause of damage to asphalt pavement. In permeable pavement, rainwater penetration into the structural layer causes the material to remain damp or saturated for extended periods, leading to a decline in material performance. Particularly during rainy conditions, the dynamic water pressure between the tires and pavement increases the likelihood of aggregate peeling and mixture loosening [12,13,14,15].

High-viscosity asphalt, with its excellent viscoelastic properties, high- and low-temperature performance, and water stability, ensures that porous asphalt mixtures have strong adhesion, water damage resistance, and good resistance to rutting. The author used polyphosphoric acid (PPA) to improve the physical and rheological properties of high-viscosity modified asphalt; its 60 °C dynamic viscosity reaches 163,735 Pa·s, far higher than the other three types of asphalt [16,17]. This has, therefore, attracted considerable academic attention. Ilyin et al. investigated the effects of polymer and solid nanosized additives on the rheological properties of asphalt pavement at an earlier time, and the results showed that the addition of polymeric modifiers (SBS) or devulcanized rubber particles substantially increases the storage and loss moduli and decreases the intensity of reduction in the storage modulus with temperature by several orders of magnitude [18]. Bahram Shirini analyzed the disparity in efficacy between rubber asphalt with different contents of rubber powder and 5% SBS modified asphalt. The findings indicated that incorporating rubber powder and SBS could enhance the material’s resilience to high-temperature deformation, moisture damage resistance, and traction, but it will reduce the water permeability of asphalt mixtures [19]. Investigations conducted by Punith et al. reveal that blending wood fibers into rubber modified asphalt improves its cohesive properties, deformation resistance, moisture integrity, and endurance against fatigue [20]. Sangiorgi et al. examined the roadway suitability of a high-viscosity asphalt blend formulated through a compound alteration of discarded rubber and SBS, noting enhancements in both its resilience to low-temperature fissuring and aerosolized particle reduction, albeit at the expense of diminished penetrability by water and resilience to lasting deformation [21]. Substances such as SBS, rubber powder, or TAFPACK-SUPER (TPS) are commonly employed to prepare high-viscosity asphalt.

Currently, the TPS modifier is the most commonly used high-viscosity modifier for high-viscosity asphalt in Japan. TPS primarily consists of thermoplastic rubber, complemented by minor quantities of resin (tackifier) and plasticizer, known for their effective performance. However, the elevated cost restricts its widespread use and application [22,23]. More researchers are now concentrating on developing high-viscosity asphalt with low cost and high performance. Raqiqa tur Rasoo1 employed recycled rubber powder and SBS to develop composite modified asphalt, and experimental findings indicate that recycled rubber powder and SBS are highly compatible, disperse evenly in the composite modified asphalt, and notably enhance its 60 °C dynamic viscosity [24]. Geng Litao used SBS and lime milk to produce high-viscosity asphalt and mixture, examining its fatigue and anti-aging characteristics, and the tests revealed that the mixture exhibits strong anti-aging and fatigue resistance [25]. Alam et al. investigated the effects of polysulfate and SBS on matrix asphalt at different concentrations. By adjusting the proportions of aromatics, resin, and asphaltene in asphalt, the 60 °C dynamic viscosity can be enhanced [26]. Zhang et al. developed high-viscosity asphalt using SBS as the main modifier, incorporating furfural essential oil as a plasticizer and sulfur as a crosslinking agent. Findings demonstrated that plasticizers facilitate the swelling and dispersion of SBS in asphalt and enhance 60 °C dynamic viscosity, and crosslinking agents help form a stable polymer network, effectively boosting asphalt’s aging resistance [27,28]. Cong et al. explored the impact of various carbon black on the performance of SBS modified asphalt, noting that carbon black enhances both the conductivity and thermal properties of asphalt, thus improving its high-temperature and anti-aging capabilities [29]. Wu et al. found that 4A zeolite boosts the aging and high-temperature stability of SBS modified asphalt, with optimal results at a 6% concentration [30].

With the development of testing technology, many researchers suggest using dynamic shear rheological testing to characterize the performance of modified asphalt. It can also provide more reliable information for studying the performance of high-viscosity asphalt. Rheology mainly studies the flow and deformation processes of materials and is a branch of mechanics. Various countries around the world classify asphalt mainly through penetration classification and viscosity classification systems [31]. With the improvement of technology and people’s understanding of rheology, the United States implemented the Strategic Highway Research Program (SHRP) in the late 1980s, and the research results on asphalt and asphalt mixtures are collectively referred to as SUPERPAVE (Super Performance Asphalt Pavement). Among them, Performance Grade (PG) is the most eye-catching [32]. Unlike previous asphalt grading and standards, the evaluation method and indicators of PG are proposed based on the road performance of asphalt binder, so it is applicable to both ordinary asphalt and modified asphalt. At present, the research methods and indicators for the rheological properties of modified asphalt are developed and changed based on PG [33]. PG classification mainly distinguishes the high- and low-temperature grades and fatigue performance of asphalt materials. Shenoy [34] corrected the rutting factor of asphalt and obtained G*/(1 − 1/(1/tanδsinδ)) as a new indicator of the high-temperature performance of asphalt. Xing investigated the dynamic shear rheological properties of different types of high-viscosity asphalt slurries, and the results showed that increasing the viscosity of modified asphalt or increasing the specific surface area of mineral powder can effectively reduce the temperature sensitivity of high-viscosity asphalt slurries to rutting factors and improve their high-temperature deformation resistance [34]. Overall, the research methods and evaluation indicators for asphalt rheological properties have mostly been continuously improved and developed based on PG, so rheological methods are more closely related to road performance than empirical testing. At present, rheological means have been widely used to study the viscoelasticity, compatibility, stability, and aging resistance of polymer modified asphalt.

However, the high-viscosity asphalt often features high polymer content, limited polymer-asphalt compatibility, and unresolved storage stability issues. This performance decline impacts the original modified asphalt. Compatibility challenges between polymer additives and asphalt continue to hinder the use of high-viscosity asphalt in engineering applications and large-scale production [35,36]. This study formulates a high-performance, cost-effective high-viscosity asphalt using orthogonal experimental design and conducts a thorough comparative analysis of its conventional performance, microstructure, and rheological properties. Analyzing the applicability and economic value of high-viscosity asphalt in porous asphalt pavements, offers insights for further performance optimization and wider application. Compared to SBS modified asphalt and rubber asphalt, the high-viscosity asphalt for porous asphalt pavements exhibits superior performance in high and low temperatures, 60 °C dynamic viscosity, and aging resistance. Additionally, it is significantly more cost-effective than commercially available high-viscosity asphalt, underscoring its considerable importance for the promotion and application of porous asphalt pavements.

## 2. Materials and Methods

### 2.1. Materials

The primary raw materials for the high-viscosity asphalt comprised SK90# matrix asphalt, manufactured in Suwon, South Korea; SBS1301, a linear type produced by Yueyang Petrochemical Plant in Yueyang of China; a tackifier primarily composed of C5 petroleum resin with a molecular weight of 1500; a liquid solubilizer of rubber oil with the model of Naphthenic acid 4010, sourced from Henan Leimo Chemical Products Co., Ltd. in Zhengzhou of China; a stabilizer of sulfur powder; and rubber powder procured from Shandong Hengfeng Rubber Powder Co., Ltd. in Binzhou, Shandong of China, featuring a fineness of 0.25 mm. The basic properties of SBS are detailed in Table 1.

This study used SK90# matrix asphalt and evaluated its basic performance according to the “Test Specification for Asphalt and Asphalt Mixtures in Highway Engineering” (JTG E20-2011) [37], as shown in Table 2.

The basic properties of rubber powder are shown in Table 3.

Rubber asphalt and SBS modified asphalt were both self-made in the laboratory. Rubber asphalt was composed of 82% matrix asphalt and 18% rubber powder; SBS modified asphalt was composed of 93.5% matrix asphalt, 4.5% SBS, 2% rubber oil, and an additional 0.15% stabilizer added to the entire asphalt system.

### 2.2. Test Method

#### 2.2.1. Conventional Performance Test of Modified Asphalt

In accordance with the procedures outlined in the ‘Highway Engineering Asphalt and Asphalt Mixture Test Procedures’ (JTG E20-2011), this study evaluated the performance indicators of asphalt, including 25 °C penetration, softening point, 5 °C ductility, 60 °C dynamic viscosity, 175 °C Brookfield viscosity, peeling rate, and segregation difference [37].

#### 2.2.2. Microscopic Analysis of Modified Asphalt

(1) Fluorescence microscopic dispersion observation test

The microscopic photography method offers a direct approach to examining the distribution and phase interface behaviors of polymers within the asphalt system, serving as an effective technique for the microscopic analysis of the modification mechanisms in polymer-modified asphalt [38,39]. Fluorescence microscopy was employed to observe the microstructure of various modified asphalts. A 0.5 g sample of asphalt was placed on a glass slide and heated to 100 °C on a heating table to ensure even spreading. Subsequently, the glass slide was positioned under an objective lens for the observation of the sample’s dispersion state.

(2) Asphalt infrared spectrum test

Using a Fourier transform infrared spectrometer (FTIR) and KBr compression method, infrared spectroscopy tests were conducted on different asphalt samples. These were conducted by taking about 100 mg of KBr in an agate mortar, grinding it into fine powder, putting it into a grinding tool, applying 8–10 tons of pressure on the tablet press, and keeping it there for 2 min. The tablet should be uniform and transparent, without cracks. The background of the tablet was collected, and a thin layer of asphalt sample was evenly applied to the KBr tablet for sample collection. During the experiment, the scanning frequency was set to 32, the resolution was 4 cm^−1^, and the scanning wavenumber range was 400 cm^−1^~4000 cm^−1^.

#### 2.2.3. Rheological Performance Test of Modified Asphalt

(1) Asphalt PG high-temperature grading test

A dynamic shear rheometer (DSR) was used for the asphalt PG testing. Test parameters for the original asphalt setting were as follows: a strain value of 12%, angular frequency of 10 rad/s, and a spacing of 1 mm between the parallel plates. Test parameters for the asphalt after aging were as follows: a strain value of 10%, angular frequency of 10 rad/s, parallel plates with an interval of 1 mm between the top and bottom, and vibration loading test at a temperature level of 6 °C. The complex modulus (G*) and phase angle (δ) were tested using a DSR. The rutting factor (G */sin(δ)) of the asphalt was calculated based on the G* and δ. According to the American SHRP research program specification, the G*/sin (δ) of the original asphalt was not less than 1.0 kPa, the G*/sin (δ) of the thin film oven test (TFOT) residual asphalt was not less than 2.2 kPa, and the |G*|·sin (δ) of the asphalt after accelerated aging using a pressure aging vessel (PAV) was not more than 5000 kPa. Through a DSR test of residual asphalt after the TFOT stage of different asphalt samples, the high-temperature grading of asphalt can be obtained [32].

(2) Low-temperature bending creep stiffness test

The low-temperature bending creep test of asphalt after the TFOT and PVA was carried out at −12 °C, −18 °C, and −24 °C by bending beam rheometer (BBR). Creep stiffness modulus S and creep rate m were measured. The low-temperature grade of the asphalt sample was determined under the condition that the creep stiffness modulus S is not more than 300 MPa and the creep rate m is not less than 0.3.

(3) Asphalt temperature scanning test

The dynamic shear rheometer was used to scan the temperature of the selected matrix asphalt and modified asphalt at a stress level of 100 Pa. The temperature scanning range was 30 °C to 80 °C. The asphalt sample was evenly applied to a 25 mm parallel plate, and the upper and lower parallel plate spacing was 1 mm for shock scanning. The complex modulus, phase angle and rutting factor of the test results were used to analyze the temperature sensitivity of different asphalts.

(4) Multiple Stress Creep Recovery (MSCR) Test

The MSCR test uses the dynamic shear rheometer to conduct repeated loading and unloading tests on asphalt at different stress levels. Two stress levels are 100 Pa and 3200 Pa, respectively, with 10 cycles for each stress level. Each cycle included a 1 s loading process for asphalt and a 9 s unloading recovery process. This study conducted creep recovery tests on different types of asphalt under 60 °C test conditions, and obtained the relationship between the time and strain of matrix asphalt and modified asphalt.

### 2.3. Orthogonal Design

To determine the optimal formulation of high-viscosity asphalt, SBS (A), tackifier (B), and solubilizer (C) were utilized as the primary raw materials. Among them, SBS and tackifier can improve the high- and low-temperature performance of asphalt, while solubilizer can adjust the compatibility and stability of the system, further improving the low-temperature performance. An orthogonal experiment with three factors and three levels was designed. The factors and levels are detailed in Table 4, utilizing an L9 (3^4) orthogonal array for the experimental design. The orthogonal experimental array is presented in Table 5.

The following evaluation indexes are used as the response of orthogonal experiment: (a) 25 °C penetration; (b) softening point; (c) 5 °C ductility; (d) 60 °C dynamic viscosity; and (e) segregation difference.

The preparation process for modified asphalt involves the following: (1) placing SK90# matrix asphalt in a constant temperature oven set at 140 °C for three hours to allow it to flow; (2) heating the matrix asphalt to approximately 180 °C in a heating sleeve, followed by the addition of SBS and tackifier while stirring, and shearing at a high speed of 4000–5000 rpm until uniform; and (3) adding solubilizer and stirring continuously for four hours to achieve a stable system.

**Figure 1:**
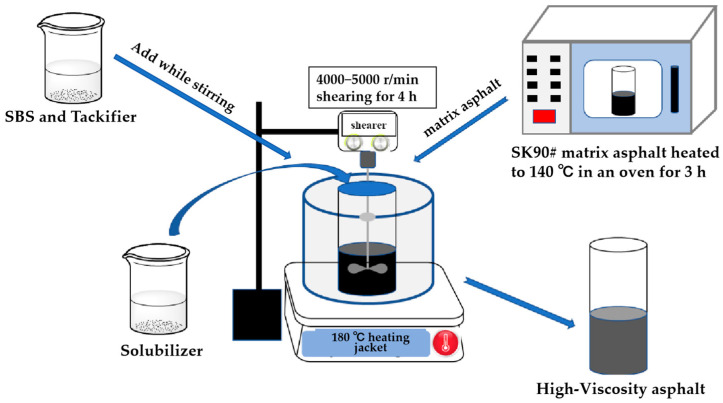
The process of preparing high-viscosity asphalt.

The process for preparing high-viscosity asphalt using SBS, tackifier, and solubilizer is illustrated in Figure 1.

## 3. Results and Discussion

### 3.1. Study on Composition Design of High-Viscosity Asphalt

Following the orthogonal experimental design, high-viscosity asphalt samples were prepared under varying factor levels. The conventional performance metrics, stability, and dynamic viscosity of high-viscosity asphalt samples across different experimental schemes were compared and evaluated, as detailed in Table 6.

An analysis of the results of the orthogonal test for high-viscosity asphalt are shown in Table 7.

Given that the range (R) serves as the primary indicator for assessing the significance of each test factor on the outcomes, a larger R value indicates a more substantial impact of the corresponding factors on the results. Conversely, a smaller R value signifies a lesser impact on the outcomes. Therefore, the range can be utilized to identify the optimal composition of asphalt formula components. The factors influencing the various evaluation indices are ranked, and the optimal scheme is determined through statistical analysis, as depicted in Table 8.

**Figure 2:**
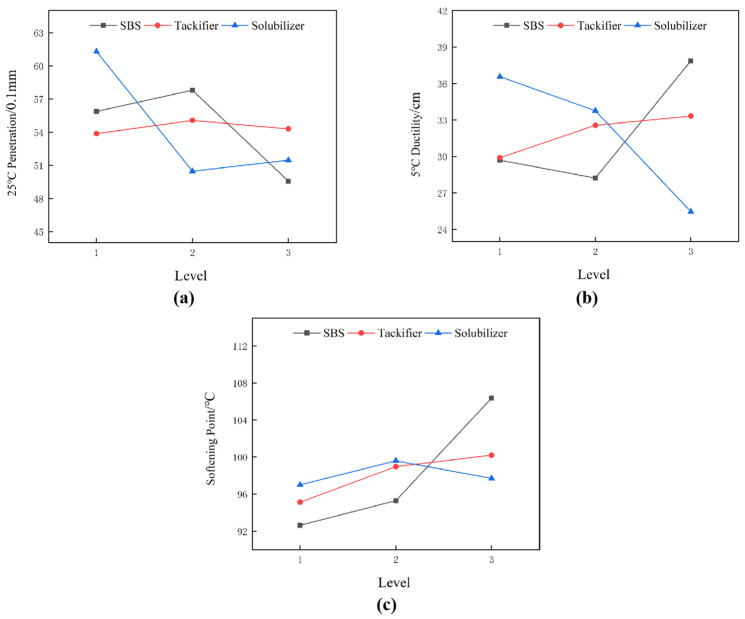
The influence of various factors on the conventional performance ((**a**) 25 °C penetration, (**b**) 5 °C ductility, and (**c**) softening point) of high-viscosity asphalt.

**Figure 3:**
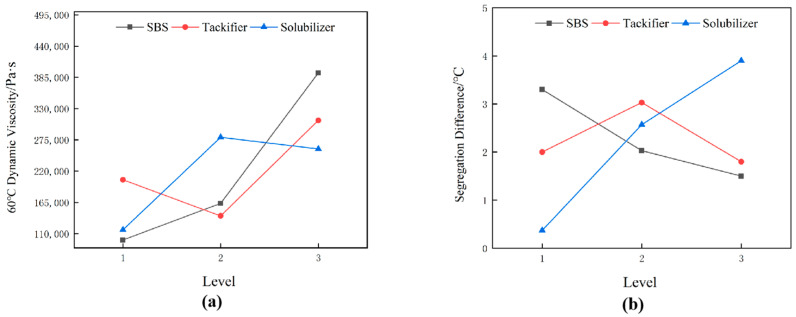
The influence of various factors on the stability and 60 °C dynamic viscosity of high-viscosity asphalt ((**a**) 60 °C dynamic viscosity, (**b**) Segregation difference).

The influence of various factors on the conventional performance of high-viscosity asphalt is shown in Figure 2. The influence of various factors on the stability and 60 °C dynamic viscosity of high-viscosity asphalt is shown in Figure 3.

Owing to the variation in optimal preparation combinations tailored to distinct indicators, the comprehensive balance method was employed to thoroughly analyze the five optimal preparation combinations previously mentioned.

(1) The influence of SBS quantity ratio (factor A) on various indicators

The influence of the SBS quantity ratio (factor A) on various indicators is evident, as depicted in Figure 2c and Figure 3a. The SBS content exhibits the most considerable variation in terms of softening point and 60 °C dynamic viscosity, signifying a notable impact on enhancing these properties of asphalt. As illustrated in Figure 2a,b and Figure 3b, the SBS range for ductility and penetration is substantial, whereas the range for segregation difference is minimal. This suggests that SBS enhances the low-temperature flexibility and viscosity of asphalt and demonstrates good compatibility with it.

(2) The influence of tackifier (factor B) on various indicators

The variation in tackifier content across different indicators is relatively narrow. As depicted in Figure 2 and Figure 3, an escalation in solubilizer content corresponds with increases in both the ductility and softening point of asphalt. At a dosage of 1%, the penetration and segregation difference exhibit their greatest values. Upon the dosage reaching 2%, the asphalt’s ductility, softening point, and dynamic viscosity attain their peak values. Despite the tackifier’s relatively minor proportion in asphalt, an optimal quantity significantly enhances asphalt performance, particularly in terms of low-temperature flexibility and dynamic viscosity.

(3) The influence of solubilizer (factor C) on various indicators

The solubilizer significantly affects the penetration, ductility, and segregation of asphalt. Solubilizer enhances the hardness of asphalt, leading to reduced softening point and ductility. Solubilizer remains in a particulate state within the asphalt. In segregation tests, higher solvent content correlates with larger differences in softening point between the upper and lower sections of the asphalt sample, and decreased storage stability. However, as illustrated in Figure 2c and Figure 3a, modified asphalt with 3% solubilizer showed a significant increase in softening point and dynamic viscosity compared to its counterpart without solubilizer. However, at a 6% concentration, there was a noticeable decline.

Higher levels of SBS and tackifier, along with lower levels of solubilizer, significantly modify asphalt, with each modifier offering complementary benefits. The high-viscosity asphalt designed for porous pavement in this study requires excellent high-temperature stability and low-temperature crack resistance. The most important indicators reflected in high-viscosity asphalt are 5 °C ductility and 60 °C dynamic viscosity. Usually, the 60 °C dynamic viscosity requires a minimum of 50,000 Pa·s to have sufficient bonding performance, and the prepared asphalt mixture must not undergo scattering and water loss. SBS and tackifier enhancers significantly contribute to the asphalt’s performance at high and low temperatures, while forming a stable system. High solubilizer content reduces compatibility with asphalt, affecting performance at various temperatures. Therefore, using high levels of SBS and tackifier, along with low levels of solubilizer, is advisable for preparing high-viscosity asphalt for porous asphalt pavement. Consequently, the optimal modifier ratios for high-viscosity asphalt are 4–5% SBS, 1–2% tackifier, and 0–3% solubilizer.

Furthermore, according to the calculation based on the commercially available raw materials, the cost of high-viscosity asphalt is about 5000 (CNY/ton), which is approximately a 30% cost savings per ton compared to commercial high-viscosity asphalts.

### 3.2. Study on the Conventional Performance of High-Viscosity Asphalt

**Figure 4:**
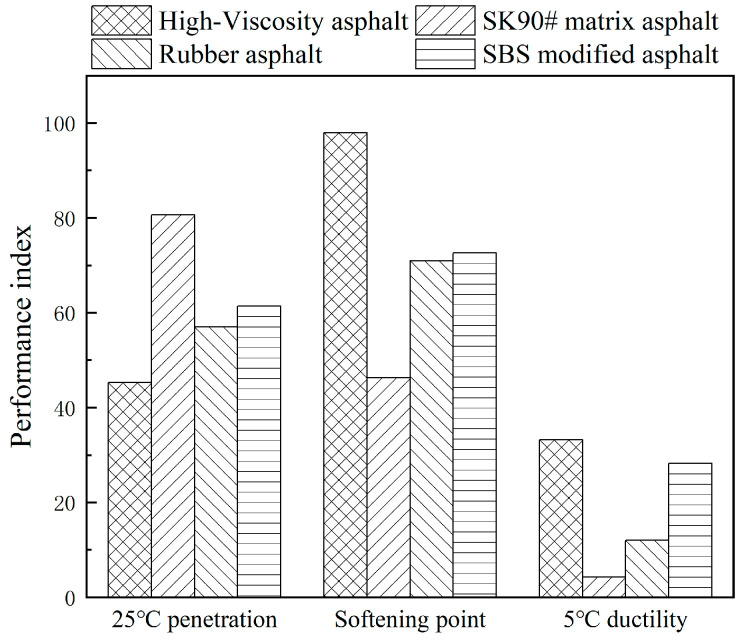
Three major indicators of each asphalt.

High-viscosity asphalt was formulated using SK90# matrix asphalt at 91%, SBS at 5%, tackifier at 2%, and solubilizer at 2%, and added stabilizer at 1.5‰ of the whole asphalt system. The conventional properties of high-viscosity asphalt, SK90# matrix asphalt, rubber asphalt, and SBS modified asphalt were compared and evaluated. The three major indicators for each type of asphalt are depicted in Figure 4, while the bonding performances are detailed in Table 9.

The three main indicators of asphalt include penetration, ductility, and softening point. Penetration reflects the relative viscosity of asphalt under certain conditions. The greater the penetration, the greater the viscosity of asphalt. Ductility reflects the low-temperature performance of asphalt, and the greater the ductility, the better the low-temperature performance of asphalt. The softening point characterizes the high-temperature performance of asphalt, and the higher the softening point, the better the high-temperature performance of asphalt.

As depicted in Figure 4, compared to SK90# matrix asphalt, high-viscosity asphalt shows a 51.7 °C increase in softening point, a 28.9 cm increase in 5 °C ductility. High-viscosity asphalt significantly enhances performance at both high and low temperatures. Considering the distribution of the three modifiers, SBS thickens the asphalt and increases its elasticity, thereby enhancing both high-temperature stability and low-temperature flexibility. High-viscosity asphalt’s performance metrics surpass those of SBS modified asphalt. Compared to SBS modified asphalt, it shows a 26.2% reduction in penetration, a 25.4 °C increase in softening point, and a 5 cm improvement in 5°C ductility. This highlights the role of tackifier and solubilizer in enhancing asphalt performance at various temperatures.

The tackifier is a high molecular weight elastomer. At high temperatures, the tackifier, being harder than the matrix asphalt, absorbs more stress with less deformation. At low temperatures, it becomes softer than the matrix asphalt, absorbing less stress but undergoing larger deformation. The solubilizer exhibits relatively high hardness, significantly contributing to the asphalt’s high-temperature performance. This enhancement of the softening point leads to an improved performance of the self-made high-viscosity asphalt at both high and low temperatures.

Higher 60 °C dynamic viscosity indicates stronger adhesion between asphalt and aggregates. The 175 °C Brookfield viscosity measures asphalt’s viscosity, with higher values indicating a higher construction temperature for the mixture, which characterizes the construction and workability of asphalt mixtures.

As indicated in Table 9, modified asphalt exhibits significantly higher 60 °C dynamic viscosity compared to SK90# matrix asphalt. The blending of asphalt and rubber powder mainly belongs to physical blending, accompanied by the physical swelling and dissolution of rubber, and will not undergo chemical reactions with asphalt, resulting in poor compatibility with asphalt [40].

Under long-term shear and swelling, SBS can be completely dissolved in the matrix asphalt, and after the addition of stabilizers, SBS is re-crosslinked. The tackifier and solubilizers in high-viscosity asphalt swell to form viscoelastic particles that adsorb in the asphalt network, further increasing the 60 °C dynamic viscosity of asphalt.

The higher the 60 °C dynamic viscosity, the more conducive it is to improving the bonding strength of high-viscosity asphalt binder and reducing the scattering and detachment of porous asphalt mixture. The adhesion of different asphalt is evaluated by the peeling rate. The larger the peeling rate, the poorer the adhesion between asphalt and aggregates. According to Table 9, the 60 °C dynamic viscosity value of high-viscosity asphalt is much higher than the specification requirement of over 50,000 pa. s. Compared with SBS modified asphalt and rubber asphalt, the 175 °C Brookfield viscosity of high-viscosity asphalt is moderate, meeting the technical requirement of less than 3.0 pa. s. The order of adhesion strength is high-viscosity asphalt > SBS modified asphalt > rubber asphalt > SK90# matrix asphalt, which shows that high-viscosity asphalt is suitable for laying porous asphalt pavement and has good construction workability.

### 3.3. Study on the Microscopic Properties of High-Viscosity Asphalt

#### 3.3.1. Observation of Fluorescence Dispersion of Modified Asphalt

**Figure 5:**
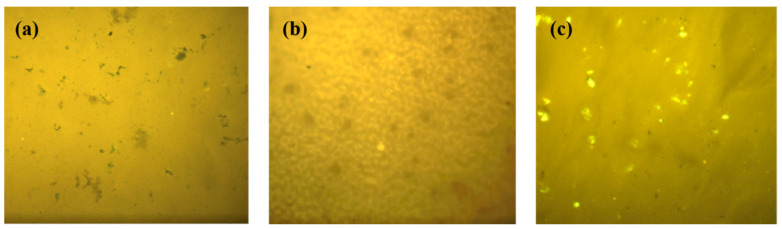
The fluorescence dispersion of different modified asphalts (**a**) rubber asphalt, (**b**) SBS modified asphalt, and (**c**) high-viscosity asphalt.

Optical microscopy is an effective auxiliary analytical device for studying the thermal stability of polymer modified asphalt. Currently, micrographs have been used as a direct method to study the distribution behavior and phase interface behavior of polymers in asphalt systems. The dispersibility of modifiers in asphalt was evaluated by directly observing the distribution of polymers in asphalt [39]. The microstructure of different modified asphalts was observed by fluorescence microscope, as shown in Figure 5.

As shown in Figure 5, the modified asphalt has a good dispersion effect. Each modified material is uniformly dispersed in the matrix asphalt without obvious agglomerates or obvious phase interfaces. Rubber particles and polymer-like particles are sheared and dissolved, forming a homogeneous and stable system.

Figure 5a shows the microstructure of rubber asphalt. After high-speed shearing, rubber particles are uniformly dispersed. After a long period of high temperature, the rubber particles absorb the oil in the asphalt and undergo swelling, while there are small dark rubber particles in the system. Figure 5b shows the microstructure of SBS modified asphalt. After long-term high-speed shear, SBS undergoes swelling, dissolution, and re-crosslinking, resulting in a clear network-like structure in the system. Figure 5c shows the microstructure of high-viscosity asphalt. The modifier is evenly distributed in asphalt and there are flocs in the asphalt system, which are the crosslinked network nodes in asphalt, increasing fusion between components, and serving as a “link”.

#### 3.3.2. Infrared Spectroscopy Analysis of Asphalt

**Figure 6:**
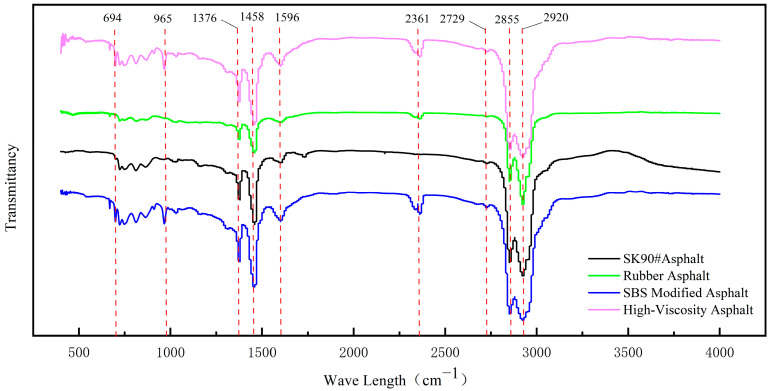
The infrared spectra of four different types of asphalt.

The FTIR spectrometer is used to conduct infrared spectroscopy tests on different asphalt samples. The infrared spectra of four different types of asphalt are shown in Figure 6.

As depicted in Figure 6, the various asphalts exhibit significant absorption peaks at 2800 cm^−1^~3000 cm^−1^, attributed to the CH_2_ stretching vibration absorption peak of alkanes or cycloalkanes. A weak vibration absorption peak at 2729 cm^−1^ corresponds to the C-H stretching vibration absorption peak. The infrared spectral analysis peaks of polymers occur in two distinct regions: 4000 cm^−1^~1300 cm^−1^ and 1300 cm^−1^~600 cm^−1^. The vibration absorption effect of functional groups is pronounced in the high-frequency region, which facilitates analysis and is significant for identifying functional groups. The low-frequency region is highly sensitive to asphalt components, and small changes can result in a strong vibration absorption effect. Therefore, this region is often referred to as the fingerprint recognition area [41,42]. At the wavelength of 2361 cm^−1^, rubberized asphalt, SBS modified asphalt, and high-viscosity asphalt exhibit vibrations, indicating the presence of asymmetric vibrations associated with accumulated double bonds or stretching vibrations of triple bonds such as –C≡C and –C≡N. The absorption peaks of SBS modified asphalt and high-viscosity asphalt at 1458 cm^−1^ and 1376 cm^−1^ are formed by the in-plane stretching vibration of –C-H in –C-CH_3_ and –CH_2_. The 1601 cm^−1^ and 1493 cm^−1^ wave lengths represent the kinetic absorption peaks of benzene nuclei. There is a significant difference in the absorption peaks between modified asphalt and matrix asphalt in the fingerprint region. In the 1000–650 cm^−1^ region, there is a benzene ring substitution zone, which produces a benzene ring skeleton (C-C) vibration and bending vibration (C-H). The 694 cm^−1^ and 757 cm^−1^ wavelengths are vibration absorption peaks of single substituted benzene rings, 965 cm^−1^ is a twisted vibration absorption peak of C=C, and 911 cm^−1^ is an out-of-plane swing vibration absorption peak of CH_2_, which is a characteristic absorption peak of polymer (SBS, thickener). It is evident that high-viscosity asphalt has a large absorption area, while rubber asphalt and matrix asphalt do not exhibit absorption peaks. The shapes of the spectra of rubber asphalt and matrix asphalt are basically the same, indicating that the modification of rubber asphalt mainly involves physical blending. SBS modified asphalt and high-viscosity asphalt have many similar characteristic absorption peaks, but no characteristic absorption peaks disappear or are newly generated, exhibiting soluble physical co-mixing and re-crosslinking, thereby significantly improving 60 °C dynamic viscosity and asphalt performance.

### 3.4. Study on Rheological Properties of High-Viscosity Asphalt

#### 3.4.1. Analysis of Asphalt Temperature Scanning Test

**Figure 7:**
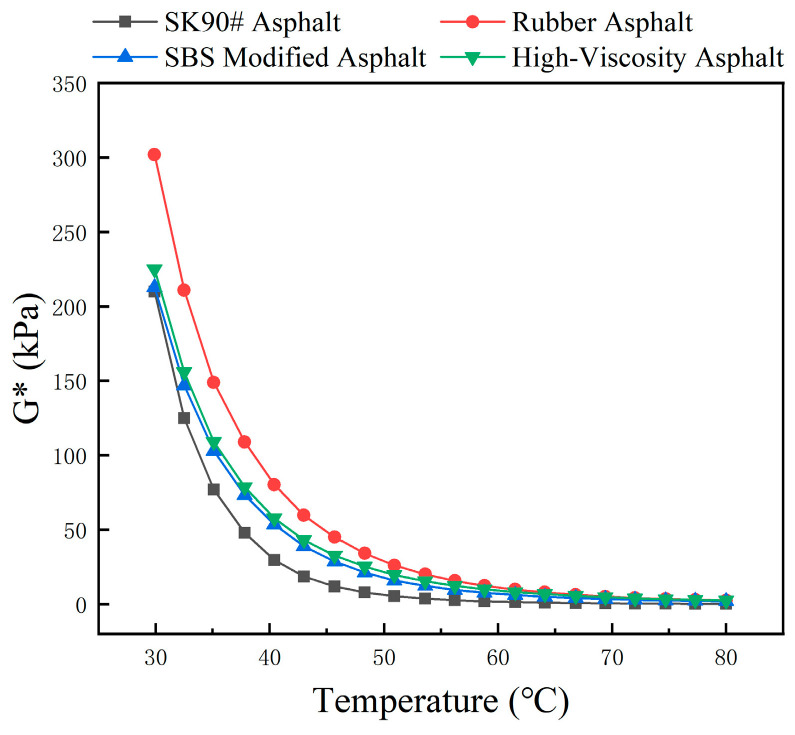
The variation in complex modulus of different asphalt with temperature.

**Figure 8:**
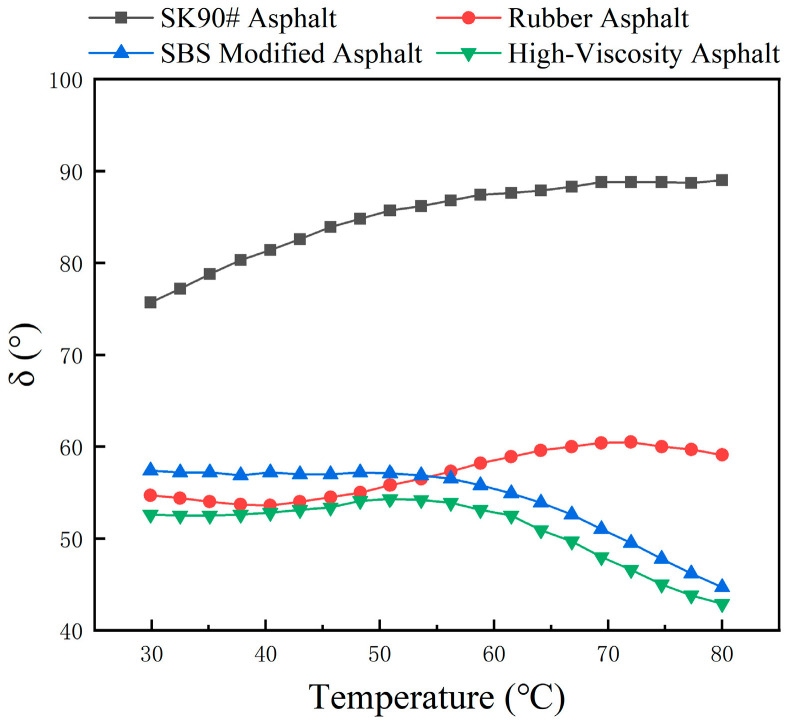
The variation in phase angle of different asphalt with temperature.

**Figure 9:**
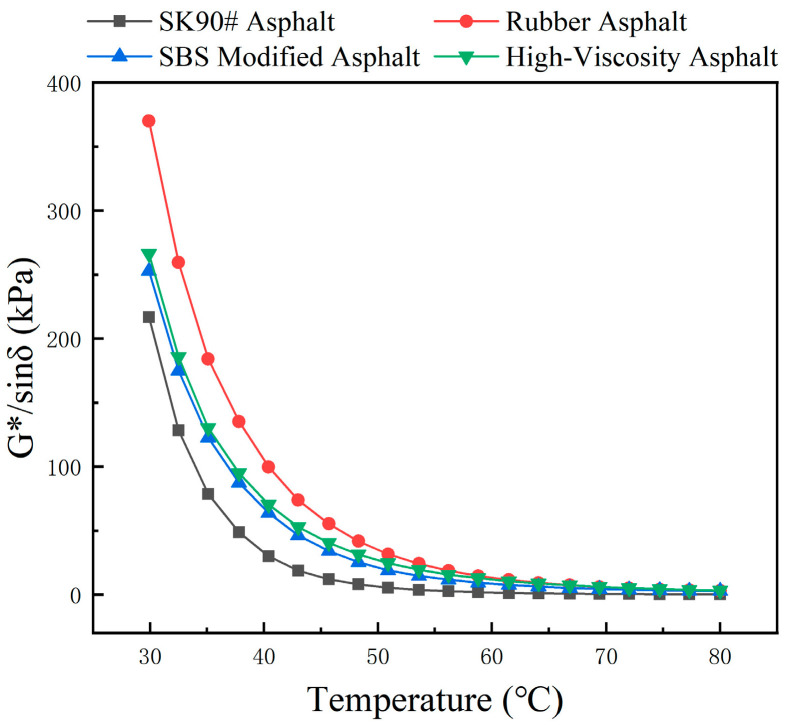
The variation in rutting factor of different asphalt with temperature.

Temperature scanning tests were conducted on SK90# matrix asphalt and three types of modified asphalt to obtain the relationship between complex modulus G*, phase angle δ, and rutting factor G*/sin(δ) with temperature, as shown in Figure 7, Figure 8 and Figure 9.

The complex modulus of four asphalt types shows similar patterns, decreasing as temperature rises. Asphalt with a higher modulus generally resists deformation at high temperatures. A larger phase angle indicates more pronounced viscosity characteristics of asphalt, reflecting strain hysteresis. Therefore, with rising temperatures, asphalt’s resistance to deformation decreases while its viscosity characteristics increase.

As depicted in Figure 7, at a consistent temperature, SK90# asphalt has the lowest modulus, while rubber asphalt has the highest among the four types. For instance, at 58 °C, the complex modulus of rubber asphalt is 6.74 times greater than that of SK90# asphalt. Adding rubber powder significantly enhances the asphalt’s modulus.

Figure 8 shows that at the same temperature, the phase angles of the three modified asphalts are smaller than that of the matrix asphalt. For example, at 50 °C, the phase angle of high-viscosity asphalt is 32.6° lower than that of the base SK90# asphalt. Modifiers significantly affect the viscoelastic properties of asphalt. With increasing scanning temperatures, the phase angle between rubber asphalt and matrix asphalt widens, indicating more viscous components. Conversely, the phase angle of high-viscosity asphalt decreases, particularly above 60 °C, showing fewer viscous components. This results in greater elasticity and improved high-temperature performance.

The rutting factor characterizes asphalt’s resistance to high-temperature deformation. Figure 9 illustrates that the rutting factor of asphalt decreases as the temperature increases [43]. Comparing four types of asphalt, SK90# exhibits lower rutting factors, suggesting that modified asphalts offer superior rutting resistance. The non-uniformity of rubber asphalt, due to the blending of rubber powder with matrix asphalt, significantly impacts its rheological test results. These test results may not accurately represent the material’s road performance. High-viscosity asphalt, which has a higher rutting factor, shows a slower decrease in this factor with rising temperatures, indicating stronger resistance to high-temperature deformation. This makes it less susceptible to temperature variations and more effective at resisting high-temperature rutting.

#### 3.4.2. Permanent Deformation Resistance of High-Viscosity Asphalt

The temperature scanning test reveals that asphalt’s viscosity and elasticity vary with temperature changes. Evaluating asphalt’s high-temperature performance based solely on viscosity and elasticity can yield unreliable conclusions. Consequently, the multiple stress creep recovery test (MSCR) is employed to more accurately assess high-temperature performance.

The creep recovery rate (R) and irrecoverable creep modulus (J_nr_) are used as high-temperature performance evaluation indicators. The temperature setting for the MSCR test is 60 °C. The creep recovery rate R and creep compliance J_nr_ can be calculated through time and strain parameters to characterize the delayed viscoelastic properties and high-temperature resistance to permanent deformation of four different asphalts. The average creep recovery rate R_3.2_, average creep compliance Jnr_3.2_, and average creep recovery rate R_0.1_ and average creep compliance Jnr_0.1_ at a stress level of 3.2 kPa and 0.1 kPa can be calculated, as shown in Table 10.

As shown in Table 10, asphalt displays varying creep recovery abilities at two stress levels. Modified asphalt’s creep deformation recovery rate is higher than that of its matrix counterpart. For matrix asphalt, this rate is negative at stress levels of 0.1 kPa and 3.2 kPa, indicating limited recovery ability. At 60 °C, the test temperature significantly exceeds its softening point, resulting in a lack of macro-level elasticity in the asphalt. During unloading, deformation often increases due to gravity. Irreparable creep compliance J_nr_ reflects asphalt’s deformation recovery strength. A higher J_nr_ indicates more irreparable deformation and reduced deformation resistance in asphalt pavement. Matrix asphalt’s irreversible creep compliance is nearly three orders of magnitude lower than modified asphalt’s. Under load, matrix asphalt undergoes the most deformation, which is almost entirely irreversible.

Modified asphalt exhibits robust deformation recovery capabilities. The creep recovery performance of SBS modified and high-viscosity asphalt are comparable, whereas rubber asphalt’s rate is the lowest. Particularly at 3.2 kPa, rubber asphalt’s recovery rate is less than half that of the other two types. High-viscosity asphalt, incorporating tackifier, solubilizer, and SBS, demonstrates high elasticity and robust resistance to high-temperature rutting.

Comparing the unrecoverable creep compliance J_nr_ of modified asphalt at different stress levels, at 0.1 kPa the order is rubber asphalt > high-viscosity asphalt > SBS modified asphalt. At 3.2 kPa, it is rubber asphalt > SBS modified asphalt > high-viscosity asphalt. This comparison reveals that high-viscosity asphalt maintains good creep recovery across stress levels, with increasing deformation resistance and excellent high-temperature rutting resistance as load levels rise.

#### 3.4.3. Analysis of Low-Temperature Crack Resistance Performance

**Figure 10:**
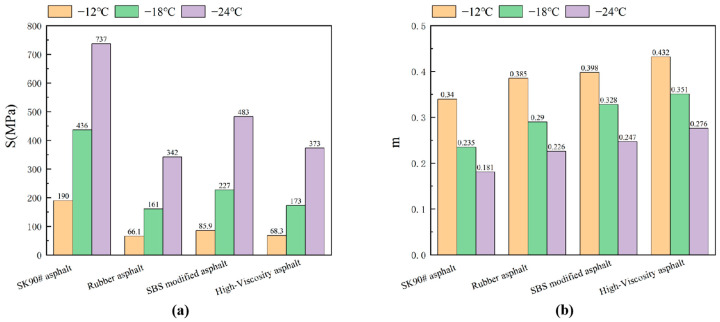
The creep stiffness modulus (S) and creep rate (m) of asphalt vary with temperature ((**a**)creep stiffness modulus, (**b**)creep rate).

The creep rate (m) reflects the asphalt’s stress relaxation capacity under lower temperature loads. A higher creep rate indicates stronger stress relaxation and improved low-temperature performance. Creep stiffness (S) measures the asphalt’s deformation resistance at lower temperatures. Higher creep stiffness suggests greater stress for the same strain, resulting in harder asphalt with reduced low-temperature crack resistance. As depicted in Figure 10, both the creep stiffness modulus S and creep rate m vary with temperature.

Figure 10 illustrates that lower temperatures result in a higher stiffness modulus and a lower creep rate, aligning with the rheological and stress relaxation properties of asphalt under these conditions. With temperature variations, the stiffness modulus of modified asphalt remains lower than that of matrix asphalt, indicating the latter’s superior rigidity at low temperatures.

Modified asphalt’s high elasticity enhances its low-temperature flexibility. The asphalt’s creep rate consistently decreases with temperature, showing a strong linear correlation. It also features a lower creep rate and effective stress relaxation. Additionally, asphalt demonstrates robust elastic recovery in low temperature conditions.

Figure 10 shows that at the same temperature, the creep stiffness modulus ranks as follows: SK90# matrix asphalt, SBS modified asphalt, high-viscosity asphalt, and rubber asphalt. Creep rates, from highest to lowest, are as follows: high-viscosity asphalt, SBS modified asphalt, rubber asphalt, and matrix asphalt. Comparing S and m values indicates that high-viscosity asphalt offers superior low-temperature flexibility. Despite its lower creep stiffness modulus, rubber asphalt’s high creep rate leads to more significant damage in low temperatures, adversely affecting road durability.

As temperature drops, m value decreases uniformly across all four asphalt types. While SBS and rubber modifications slightly increase asphalt’s creep rate, high-viscosity modifiers have a more pronounced effect. Thus, the combined use of viscosity enhancers and solvents in high-viscosity asphalt maximizes the benefits of both, boosting stress relaxation and improving low-temperature performance.

#### 3.4.4. Analysis of PG Results for High-Viscosity Asphalt

Through the DSR test of residual asphalt after the TFOT stage of different asphalt samples, the high temperature grading of asphalt can be obtained. The PG results for four types of asphalt are shown in the Table 11.

Modified asphalts exhibit improved high-temperature grades over matrix asphalt. Rubberized and SBS modified asphalts have a high-temperature grade of 76 °C, three degrees higher than matrix asphalt, while high-viscosity asphalt reaches 88 °C, five degrees higher. Following short-term aging, modified asphalts’ high-temperature grades typically decrease due to their viscosity, which impedes smooth flow and uniform film formation during the thin film oven aging process, unlike matrix asphalt. Additionally, in long-term high temperatures, asphalt’s asphaltene content increases, while its resin, aromatic, and saturated components decrease. This change, coupled with repeated loading, leads to irreversible deformations due to increased viscous components.

Both matrix and rubber asphalts are graded at PG-12 °C for low temperatures, with a sharp increase in creep stiffness modulus as temperatures drop. This results in a higher creep rate and reduced low-temperature crack resistance. Despite both SBS and high-viscosity asphalt having a low-temperature grade of PG-18 °C, high-viscosity asphalt outperforms SBS in creep stiffness and rate, offering superior stress relaxation and flexibility at low temperatures, thus enhancing crack resistance. The synergistic use of viscosity enhancers and solvents in self-made modified asphalt combines the strengths of both, significantly improving its low-temperature capabilities.

The analysis indicates that all three modified asphalt types enhance both high- and low-temperature performance of asphalt. High-viscosity asphalt, in particular, shows a more pronounced improvement in these areas. Using high-viscosity modifiers yields the best results in temperature performance enhancements. This modification also significantly boosts rut resistance, leading to optimal overall performance.

## 4. Conclusions

The three factors and three levels orthogonal experiment was used to explore the effect of raw material ratio on the performance of modified asphalt, and the optimal ratio range was determined based on the relevant technical indicators of porous asphalt pavement. A high-viscosity asphalt was prepared according to the optimal ratio, and matrix asphalt, rubber asphalt, and SBS modified asphalt were selected as control groups. The microstructure, conventional properties and rheological properties of different types of asphalt were compared and analyzed. The primary conclusions are summarized as follows:

(1) The optimal quantity ratio of high-viscosity asphalt is determined to be SBS content of 4%–5%, tackifier content of 1–2%, solubilizer content of 0–3%, and stabilizer content of 0.15%.
(2) Compared with SBS modified asphalt, rubber asphalt, and matrix asphalt, the softening point, 5 °C ductility, and 60 °C dynamic viscosity of high-viscosity asphalt were significantly improved, while the 175 °C Brookfield viscosity was equivalent to SBS modified asphalt. In particular, the 60 °C dynamic viscosity reaches 383,180 Pa·s. Rheological tests indicate that the high- and low-temperature grade of high-viscosity asphalt reaches 88–18 °C, and high-viscosity asphalt has the best high-temperature resistance to permanent deformation and low-temperature resistance to cracking.
(3) The components evenly dispersed and the performances were enhanced with chemical and physical modification. The SBS and thickener exhibit soluble physical co-mixing and re-crosslinking, thereby significantly improving asphalt performance.
(4) The comprehensive performance of high-viscosity asphalt has been greatly improved and can save about 30% in costs compared to commercially available high-viscosity asphalt, which is conducive to the promotion and application of porous asphalt pavement.

## Figures and Tables

**Table 1:** Basic properties of SBS.

Properties	Unit	Value
S/B ratio	/	30/70
Extender oil content	/	0
Modulus at 300%	MPa	≥2.2
Tensile strength	MPa	≥16.0
Elongation at break	%	≥700
Tensile set at break	%	≤40
Melt flow rate	g/10 min	0.5~2.5
Molecular weight	/	100,000

**Table 2:** Basic properties of SK90# matrix asphalt.

Properties		Technical Requirements	Value
Penetration (25 °C, 100 g, 5 s) (0.1 mm)		80.6	80~100
Softening point (°C)		46.3	≥42
Ductility (5 cm/min, 10 °C) (cm)		>100	30
60 °C dynamic viscosity (Pa·s)		145.2	≥140
After TFOT	Mass loss (%)	0.011	≤±0.8
	Penetration ratio (25 °C) (%)	84.7	≥50
	Ductility (5 cm/min, 10 °C) (cm)	9.6	≥6

**Table 3:** Basic properties of rubber powder.

**Component**	Ash	Rubber hydrocarbon	Fiber	Carbon black
**Dosage**	3%	45%	0.08%	38%

Note: Fibers are generated during the process of manufacturing waste rubber powder from waste tires, generally consisting of polyamide fibers and polyester fibers and accounting for about 5% of the total weight of tires.

**Table 4:** Factor levels of asphalt modifier formulation.

Code	Influencing Factor	Level		
		1	2	3
A	Mass ratio of SBS	3%	4%	5%
B	Mass ratio of tackifier	0	1%	2%
C	Mass ratio of solubilizer	0	3%	6%

**Table 5:** Orthogonal experimental design table.

Serial Number	Factors		
	A	B	C
1	1	1	1
2	1	2	2
3	1	3	3
4	2	1	2
5	2	2	3
6	2	3	1
7	3	1	3
8	3	2	1
9	3	3	2

**Table 6:** Orthogonal experimental results of high-viscosity asphalt.

Serial Number	A (%)	B (%)	C (%)	25 °C Penetration (0.1 mm)	5 °C Ductility (cm)	Softening Point (°C)	60 °C Dynamic Viscosity (Pa·s)	Segregation Difference (°C)
1	3	0	0	62.1	32.1	90.8	44,550	0.5
2	3	1	3	51.8	32.3	93.3	85,915	5.1
3	3	2	6	53.7	24.7	93.8	166,305	4.3
4	4	0	3	54.2	28.1	93.3	146,590	1.9
5	4	1	6	55.4	22.2	98	187,675	3.8
6	4	2	0	63.8	34.4	94.6	155,840	0.4
7	5	0	6	45.3	29.5	101.3	423,090	3.6
8	5	1	0	58.0	43.2	105.6	150,110	0.2
9	5	2	3	45.4	40.9	112.2	605,890	0.7

**Table 7:** Analysis of orthogonal test results for high-viscosity asphalt.

Analysis Indicators		Factor A	Factor B	Factor C
Penetration 25 °C	K_1_	55.87	53.87	61.30
	K_2_	57.80	55.07	50.47
	K_3_	49.57	54.30	51.47
	R	8.23	1.20	10.83
5 °C ductility	K_1_	29.7	29.90	36.57
	K_2_	28.23	32.57	33.77
	K_3_	37.87	33.33	25.46
	R	9.64	3.43	11.11
Softening point	K_1_	92.63	95.13	97.0
	K_2_	95.30	98.97	99.6
	K_3_	106.37	100.2	97.7
	R	13.74	5.07	2.60
60 °C dynamic viscosity	K_1_	98,920	204,745	116,835
	K_2_	163,370	141,235	279,470
	K_3_	393,030	309,345	259,020
	R	294,110	168,110	162,630
Segregation difference	K_1_	3.30	2.00	0.37
	K_2_	2.03	3.03	2.57
	K_3_	1.50	1.80	3.90
	R	1.80	1.23	3.53

Note: K_i_ is the arithmetic mean value of the corresponding test data when i is taken as the factor level of any column, i = 1, 2, 3; the range R is the maximum difference of K_i_ at this level.

**Table 8:** Comprehensive analysis table of orthogonal experiments.

Evaluating Indicators	Ranking of Impact Levels	Best Scheme
25 °C penetration	C > A > B	A_2_B_2_C_1_
5 °C ductility	C > A > B	A_3_B_3_C_1_
Softening point	A > B > C	A_3_B_3_C_2_
60 °C dynamic viscosity	A > B > C	A_3_B_3_C_2_
Segregation difference	C > A > B	A_3_B_3_C_1_

**Table 9:** The bonding performances of asphalt.

Item	High-Viscosity Asphalt	SK90# Matrix Asphalt	Rubber Asphalt	SBS Modified Asphalt
60 °C Dynamic viscosity/Pa·s	383,180	145	16,700	47,100
175 °C Brookfield viscosity/Pa·s	1.593	0.083	3.418	1.245
Peeling rate/%	22.3	57.4	40.1	36.4

**Table 10:** Calculation results of creep recovery rate and irreversible creep compliance.

Index	SK90# Matrix Asphalt	Rubber Asphalt	SBS Modified Asphalt	High-Viscosity Asphalt
R_0.1_	−0.0081	0.8573	0.9905	0.934
R_3.2_	−0.0127	0.4177	0.9781	0.9693
J_nr0.1_(kPa^−1^)	30.285	0.3936	0.0242	0.183
J_nr3.2_(kPa^−1^)	33.42	1.5822	0.0891	0.0863

**Table 11:** The PG results for four types of asphalt.

Asphalt Type	PG (°C)	Creep Stiffness (MPa)	Creep Rate	Original Asphalt Rutting Factor (kPa)	TFOT Residue Rutting Factor (kPa)
SK90# matrix asphalt	58~12	190	0.340	2.30	4.76
Rubber asphalt	76~12	66.1	0.385	3.48	3.71
SBS modified asphalt	76~18	227	0.328	2.82	2.71
High-viscosity asphalt	88~18	173	0.351	2.36	2.40

## Data Availability

Data are contained within the article.
